# The Reason of the Faith That Is in Me

**Published:** 1884-03

**Authors:** Samuel Swan

**Affiliations:** New York


### THE REASON OF THE FAITH THAT IS IN ME.

SAMUEL SWAN, M. D., NEW YORK.

For some years I have been the recipient of notices (far from flattering) in the journals on account of alleged departures from the teaching and practice of Hahnemann, especially because of my discovery that "morbific products would cure the disease which produced them if given in a high potency," and also because of my formula of potentization of remedies, in that I had no right to call them centesimal because they were not made in accordance with Hahnemann's formula.

In my pamphlet entitled "Nosodes and High Potencies," I gave an account of cures, of cases that were not likely to recover without medicine, that were sufficient to carry conviction to any unprejudiced mind that those were remedies of great value. Instead of an honest acceptance of the facts, and admitting that the remedies were worth proving, even if the knowledge of their therapeutic powers had come in through a back door, an attempt was made to belittle them as insignificant and unworthy of notice. Now I propose to show that I have the authority of Hahnemann on my side in both counts of the indictment preferred against me, and also that *he was the original discoverer and promulgator of the so-called "heresy."*

First, as to potencies: According to Hahnemann's formula for potentizing, it would require ten hundred drops of water to one drop of tincture to make the tenth centesimal potency, filling the vial with one hundred drops of water, and emptying each time. But because I allowed the entire ten hundred drops to flow in and out of the vial in a continuous stream, and called it the tenth centesimal potency, I was denounced in the journals and in the lecture-room.

Hahnemann states (*Chronic Diseases*, Vol. I, page 119) as follows: "*In order to obtain it* (the best mercurial preparation) *as perfect as possible and with the least trouble* (for the greatest simplicity should be observed in preparing homeopathic remedies), *it is better to follow the method which I shall indicate below.* (Italics are mine.) *Take a grain of the purest liquid Quicksilver and triturate it* for *three hours* with *three hundred grains of Sugar of Milk, taking one hundred grains at a time and triturating them for an hour. In this way you obtain the millionth degree of trituration,*" or the third centesimal potency. Observe, that he does not say, take *one* grain of the first one hundred grains after triturating and add one hundred grains of Sugar, as he afterward proposed, *but he added one hundred grains of Sugar each time to the quantity already triturated.*

Now, if it is admissible to add three hundred grains of Sugar to one grain of liquid Quicksilver, and after triturating it for three hours, in sections of one hundred grains at a time, and calling it the one millionth degree of trituration, equivalent to the third centesimal potency, why is it not admissible for me to take one grain of a drug, and ten hundred grains of sugar, and triturating them with such a quantity of sugar in sections as is most convenient, to call it the tenth centesimal potency? And if the rule holds good with grains and triturations, why not with drops and dilutions?

I have endeavored to state the above without any distortion of language or meaning, and I wish to call attention to the fact that Hahnemann does not denounce or condemn any other mode of preparation, but says that this is the "*better*" way, and by this mode it is obtained "*as perfect as possible*," but *does not* say it was the *best*.

Every little while some one attempts to prove by figures that my preparations are very low potencies, and that those made by other processes are much higher. Hahnemann, in his *Lesser Writings*, says: "*There can be no standard for measuring the degree of dynamic potency, except the degree of reaction of the vital force.*" This is the experience of all who have ever used high potencies, and they were not induced to use them on account of their notation, or from any theory of their action, but because experience had taught them that the higher the potency the better and quicker they acted. As my mode of potentizing and notation were published, so that all might see that there was no cryptoform manipulation in their manufacture, every physician that uses them has a definite idea which potency to prescribe in a given case, from an experience which he would be compelled to have before using high potencies made as Hahnemann subsequently advised.

High potencies are not a *necessity*, but a very valuable *accessory* in the healing art. Hahnemann did not believe in attempting to gauge the value of a potency by mathematical calculation. On page 820, *Lesser Writings*, he refers to "those arithmeticians who seek to limit nature and render her contemptible by applying their multiplication table to the phenomena of her illimitable forces."

Hahnemann considered succussion important in the preparation of remedies, in order to procure not only a thorough admixture, but subsequent disintegration of the molecules, even after they had passed that stage where the degree of dynamic potentization rendered measuring impossible. He justifies this conclusion by further stating, that the reason why one drop of tincture could not medicate the Lake of Geneva or a hogshead of water was simply because of the impossibility of a thorough and complete mixture and impregnation.

As regards morbific products (isopathic), Hahnemann says (*Chronic Diseases*, volume I, page 195): "In the subsequent list of antipsoric remedies no isopathic remedies are mentioned." The reason he gives is, "that their effects upon the *healthy* organism have not yet been *sufficiently* ascertained." It would seem from this *that he had these isopathic remedies, had potentized them* (see reference to the various degrees of potency of Psorin, page 195), *had used them on the sick, had found how valuable they were, had partially proved them on healthy organisms*, but not so *thoroughly* as to warrant his giving them to the profession.

He thus disposes of isopathy. On page 196, *Chronic Diseases*, he says: "*I call Psorin a homœopathic antipsoric, because if the preparation* (potentization) *of Psorin did not alter its nature to that of a homœopathic remedy, it never could have any effect upon an organism tainted with that same identical virus.*"

The corollary is inevitable. The potentization of the isopathic product *makes it homœopathic to the disease which produces it*, and it cannot have any effect on that disease *till* potentized, but *when* potentized it does have an effect, and the effect must be homœopathic, and therefore of necessity a curative effect, or, in other words, "*Morbific matter will cure the disease which produces it if given in the highest attenuations.*"

*Had not Hahnemann tried morbific products empirically on those sick of the diseases which had produced those products, he would not have said that unless these were so altered by potentization they never could "have any effect on an organism tainted with that same identical virus.*"

I withdraw my claim to the discovery of the LAW in favor of Hahnemann, and only claim that others, as well as myself, have verified the truth of his great discovery.

As there is a tendency in the human mind to range at will where there is no law, Hahnemann formulated his vast experience in a certain mode of preparing remedies, in a certain mode of proving remedies, in a certain manner of examining patients, and in a certain mode of selecting and administering the remedy, knowing if those rules were closely followed, and united to a careful and thorough study of the action of drugs as revealed by their provings, greater success would result than from any other mode, as all of his faithful followers have demonstrated.

Hahnemann did not make public any remedy, no matter how much *he* knew about it, till it had been proved according to the rule he had laid down, but in the same volume he gives some of the toxical effects of Psora, Syphilis, and Sycosis, which were probably the "keynotes" from which he prescribed for those "tainted with that same identical virus." He evidently believed that later the problem of the use of these morbific products would be solved, as he says in the foot-note of ¶ 56, page 194, in the *Organon*, "but supposing this were possible, and it would deserve the name of a valuable discovery, etc." The problem *is* solved by using those products in the *high potencies*.

In my researches in this field, I had no object in view other than to enlarge the armamentarium of our school with valuable remedies, ascertaining their powers, by prescribing them in accordance with the well-known toxical effect of the miasms, and by clinical experience, to interest observing physicians, that they might be induced to prove them. The remedies could only cure homœopathically, and as they *do* cure, it shows that they were homœopathic in those cases narrated. All I ask is an unprejudiced examination of this statement, and if I am in error, show me wherein.

